# The Chemical, Rheological, and Sensorial Characteristics of Arabic Bread Prepared from Wheat-Orange Sweet Potatoes Flour or Peel

**DOI:** 10.3390/foods12081658

**Published:** 2023-04-15

**Authors:** Haiam O. Elkatry, Hossam S. El-Beltagi, Khaled M. A. Ramadan, Abdelrahman R. Ahmed, Heba I. Mohamed, Hala Hazam Al-Otaibi, Mohamed A. A. Mahmoud

**Affiliations:** 1 Food and Nutrition Science Department, Agricultural Science and Food, King Faisal University, Al-Ahsa 31982, Saudi Arabia; helkatary@kfu.edu.sa (H.O.E.); arahmed@kfu.edu.sa (A.R.A.); hhalotaibi@kfu.edu.sa (H.H.A.-O.); 2 Home Economics Department, Faculty of Specific Education, Ain Shams University, Abassia, Cairo 11772, Egypt; 3 Agricultural Biotechnology Department, College of Agriculture and Food Sciences, King Faisal University, Al-Ahsa 31982, Saudi Arabia; 4 Biochemistry Department, Faculty of Agriculture, Cairo University, Gamma St, Giza 12613, Egypt; 5 Central Laboratories, Department of Chemistry, King Faisal University, Al-Ahsa 31982, Saudi Arabia; kramadan@kfu.edu.sa; 6 Department of Agricultural Biochemistry, Faculty of Agriculture, Ain Shams University, Hadayek Shobra, Cairo 11241, Egypt; 7 Biological and Geological Sciences Department, Faculty of Education, Ain Shams University, Cairo 1575, Egypt; hebaibrahim79@gmail.com

**Keywords:** orange sweet potato, food wastes, food fortification, antioxidant activity, sensory analysis, Arabic bread

## Abstract

The current study investigates the feasibility of preparing Arabic bread from wheat flour, sweet potato flour, or peeled sweet potatoes based on the nutritional values, technological characteristics, and sensory properties of the final products. First, we analyzed the proximate, elemental, total and individual phytochemical compositions of the raw materials and bread samples. The analysis showed that potassium, calcium, and phosphorus were higher in peels than pulp, in the same manner to the total phenolics, flavonoids, and anti-radical scavenging activities. Phenolic acids and flavonols were quantified, where *p*-coumaric, feruloyl-D-glucose, eucomic, gallic, and ferulic acids were measured as major phenolic acids in either peels or pulp flours, and their quantities were higher in the peels. Furthermore, we evaluated the effects of wheat substitution on the properties of the dough blends and their final bakery. The results indicated that the fortified samples’ nutritional and rheological properties were significantly improved, while their sensory qualities were comparable to those of the control. Thereby, the fortified dough blends presented higher dough stabilities, indicating a wider range of applications. Additionally, after the heat treatment, the fortified breads maintained significantly higher total phenolic, flavonoid, anthocyanin, and carotenoid contents, and total antioxidant activities, implying their accessibility for humans upon consumption.

## 1. Introduction

A tubular agricultural crop known as the sweet potato (SP) is tolerant to a variety of abiotic stresses and can thus adapt to a variety of different climate conditions while producing a high yield [1]. It has been ranked as the seventeenth most important crop in terms of value, with a total production of 88.8 million tons in 2021, where Asia and Africa account for ≈95% of the total world production (https://www.fao.org/faostat/en/#data/QCL accessed on: 1 March 2023).

The SP roots are more abundant in carbohydrates, various provitamins and vitamins, and minerals compared to other vegetables and grains [2,3,4]. Furthermore, they contain phytochemicals including polyphenols, flavonoids, anthocyanins, and carotenoids [1,5,6]. These constituents were reported to have high antioxidant and anti-inflammatory capacities and presented health benefits, e.g., protection against colorectal cancer, increased glucose/insulin tolerance for diabetic people, and improved visual acuity [7,8,9,10]. Specifically, SPs are rich in phenolic acids, including chlorogenic (caffeoylquinic), caffeic, *p*-coumaric, and their derivatives [11,12]. These compounds were reported to suppress the growth of foodborne microorganisms, have anti-mutagenic effects, and prevent the proliferation of cancer in humans [13,14,15].

The continuous breading programs led to the development of varieties that differ in color ranging from white to red or purple [1,3,4,16]. Studies indicated that the levels of macro components, secondary metabolites, and antioxidant activity were significantly different in relation to the differences in their colors, though SP roots with a dark color have limited technological utilization in some products [1,3,4,16,17].

Industry requires the peeling of SPs to increase the quality of SP flour [16]. Depending on the peeling method, SPs could lose up to 40% of the raw material using the old lye technologies [13]. It has been confirmed that SP peels are rich in minerals and bioactive substances such as polyphenols and dietary fiber. Thus, the peeling process decreases the health benefits of the final product and contributes significantly to agri-food waste worldwide [13]. In this regard, Cui and Zhu [16] have investigated the mineral contents of unpeeled SPs and compared their results with the peeled SPs belonging to the same varieties from the previous study of Waramboi et al. [17], and the study concluded that non-peeled SPs have higher mineral contents compared to the peeled ones [16,17].

One of the oldest and most popular foods in the world, bread, is typically made from wheat flour, salt, and water, either with or without a leavening agent. White bread contains macronutrients including carbohydrates, protein, lipids, and fibers, but is deficient in some essential amino acids, micronutrients, and bioactive compounds [18,19]. The majority of the efforts being made by the food manufacturing sector and food researchers at the moment are focused on meeting consumer demands and expectations, who increasingly demand foods that enhance their nutritional status, health, and well-being [20,21,22]. A promising method to improve the nutritional value of breads while producing a healthier product and a source of innovation for the food industry is to substitute other tubular-based and nutrient-rich components for refined wheat flour while making bread [23,24,25]. Moreover, there are continuing efforts to establish and apply different utilization methods for agri-food wastes rich in nutrients and bioactive molecules as supplements for different food concepts, including bakery products [24,26,27,28,29,30,31]. In this regard, the current manuscript aims to analyze the macronutrients, minerals, phytochemicals, and antioxidant activity of orange sweet potato peels (SPPs) and sweet potato flour (SPF). We further assessed, for the first time, the feasibility of using SPPs and SPF in supplementing Arabic bread at different addition ratios to improve its nutritional value while retaining the high sensorial and rheological qualities of the final product and validated our results using chemometric methods.

Antioxidant capacity methods are widely used to assess the efficiency of pure compounds and extracts to scavenge reactive oxygen species. These tests include 2,2-diphenylpicrylhydrazyl (DPPH) and 2,2′-azino-bis(3-ethylbenzothiazoline-6-sulfonic acid) (ABTS) antiradical scavenging methods [32,33]. For the DPPH test, both the antiradical efficiency (AE) and antioxidant activity index (AAI) have been introduced to cope with the lack of standardization across the reported methods in the literature [34,35]. Although the ABTS test has a similar mechanism and drawbacks to the DPPH test, no previous reports found it necessary to use AE or AAI values for standardization of this test. Thus, we also set out to test the possibility of introducing methods of standardization for the ABTS test, similar to the DPPH test, based on the Trolox equivalent antioxidant capacity (TEAC).

## 2. Materials and Methods

### 2.1. Raw Materials and Doughs Preparation

Orange sweet potatoes used in this study were obtained from the local market in Hofuf, KSA. Fresh samples were thoroughly washed, peeled, washed again, drained, and chipped. Potato chips and peels were then oven-dried in a laboratory oven (105 °C, 1–3 h). Dried samples were milled in the laboratory mill for 2 min at 35,000 rpm (Huge Grinder Model No. E03407, Beijing, China) and finally sieved to a fine powder.

The ingredients for the dough, sucrose, instant dry yeast, and wheat flour (72% extraction) were purchased from the local market in Hofuf, Saudi Arabia. The mixing recipes for these materials as well as the addition ratios of sweet potato flour (SPF) or peels (SPPs) are presented in Table 1. These dough blends were prepared using a mixer with a spiral blade for five minutes in a Kitchen Aid Professional mixer. Furthermore, the rheological properties of dough blends were examined with the Brabender Farinograph according to A.A.C.C [36].

### 2.2. Preparation of Arabic Bread

The dough blends were placed in a proof cabinet at 30 °C and 75–80% relative humidity for 45 min for fermentation. They were then punched down to remove gases, proofed again for a further 45 min, turned into loaves, and finally baked in an electric-wheel oven for 1.5 min at 350 °C [37]. In total, three independent processes were performed to produce a total of three replicates.

### 2.3. Determination of Color

The color of the Arabic bread loaves was determined using a Minolta Colorimeter (CR 200 Japan), and the readings were expressed in Hunter values for lightness (L*), redness (a*), and yellowness (b*) according to the tristimulus color system [38].

### 2.4. Sensory Analysis

Sensory evaluation of samples was performed by a trained sensory panel, no known illness, recruited from the Food Science and Nutrition Department, King Faisal University, Al-Ahsa, Saudi Arabia (four females and six males with ages ranging between 3 and 5 years old). The description of the sensory room, the environmental conditions, and sample coding and presentation are fully described in our previous studies [21,30]. During the assessment, panelists were asked to evaluate the presented samples based on their taste, chewability, texture, aroma, color, roundness, crumbliness, appearance, and overall acceptance on a 10-point numerical scale from strongly disliking (0) to strongly liking (10). Scores were statistically analyzed, and their averages were plotted on a spider-web diagram.

### 2.5. Chemical Proximate Composition

The three independent bread samples from each treatment were combined to create a single uniform sample prior to chemical analysis. The unified samples were then subjected to each of the chemical measurements in triplicates.

According to the method outlined in A.O.A.C [39], the following substances were measured: moisture, total nitrogen, total fat, crude fiber, ash, total carbohydrates, and reducing sugars or starch. The crude fiber was identified utilizing a fully automated technique (FOSS-Fibertic 8000, Hilleroed, Denmark). According to Jayaraman [40], free amino acids were measured in an 85% ethanolic extract of powdered samples using the ninhydrin reagent (ACS-Sigma Aldrich, St. Louis, MO, USA) at 570 nm using a spectrophotometer (Thermo Scientific Evolution 350 UV-Vis spectrophotometer, Waltham, MA, USA). L-lysine (10–100 μg/mL) was used for the standard calibration curve.

### 2.6. Determination of Trace Element Levels

#### 2.6.1. Microwave Digestion

According to Marin et al. [41], 1.0 g of the powdered sample was digested in a closed microwave digestion/extraction (SINEO-MDS-6G SMART, Shanghai, China) system with either 2 mL of H_2_O_2_ (30%, ultra-pure for AA, Carlo-Erba, France) or 8 mL of HNO_3_ (65%, for ICP, Sigma-Aldrich, St. Louis, MO, USA). The mixtures were refrigerated, filtered, and diluted with ultrapure water from the Milli-Q system (Millipore, Molsheim, France) to 25 mL before ICP-OES analysis.

#### 2.6.2. Trace Element Levels Determination by ICP-OES

To maintain plasma and carrier gas, an autosampler AS 93-plus with argon (purity > 99.995%) was utilized. Shimadzu ICP-OES (9820 Series-Tokyo, Japan) equipped with an ASX-280 (Tokyo, Japan) autosampler was utilized to measure the amounts of trace elements. All the elements that were evaluated (Ca, P, K, Mg, Na, Cd, Se, Cr, Cu, Fe, Ni, and Zn) were included in an individual elemental standard solution from Sigma-Aldrich, St. Louis, MO, USA, that was 1000 mg/L to calibrate. The calibration range was (0–10 mg/L) for each element.

### 2.7. Extraction of the Lipophilic Fraction

Each sample (0.5 g) was extracted twice with 5 mL of 100% n-hexane (Sigma-Aldrich, St. Louis, MO, USA) to produce a lipophilic-free sample. The extracted samples were evaporated under low pressure.

### 2.8. Extraction and Determination of Phytochemical Fractions

A free phenolic fraction was obtained by extracting the total phenolics from the lipophilic free residuals using 5 mL of acetone/water/acetic acid (70/29.5/0.5 (*v*/*v*/*v*); AWA) for 2 h at 25 °C with shaking (Witeg shaking water bath, Wertheim, Germany). The supernatant was collected after centrifugation at 10,000× *g* for 20 min at 20 °C (HERMEL -Z36HK—, Wertheim, Germany), and the extraction was carried out once more. Each supernatant was combined. The extract was first reconstituted in 10 mL of 100% acetonitrile after being evaporated under low pressure.

#### 2.8.1. Determination of Total Phenolic Concentration

With a few modifications, the total phenolic content was calculated as per Goffman and Bergman [33]. A mixture of 0.1 mL of the reconstituted fraction in acetonitrile; 0.50 mL of deionized water; 0.25 mL of 20% Folin-Ciocalteu reagent (Fluka, Milwaukee, WI, United States); and 0.5 mL of 0.5 M ethanolamine was added after five minutes. The optical density of the mixture was read at 760 nm using a Thermo Scientific Evolution 350 UV-Vis spectrophotometer (Waltham, MA, USA) after 90 min of color development at room temperature. The amount of total phenolic in a sample was expressed as mg of gallic acid (GA)/100 g sample (DW).

#### 2.8.2. Determination of Total Flavonoid Concentration

The total flavonoid content was assessed using a spectrophotometric method outlined by Chang et al. [42], and the total flavonoid content was measured. One ml of deionized water and 0.075 mL of 5% sodium nitrite (*w*/*v*) were combined with 0.25 mL of the reconstituted fraction in acetonitrile. The mixture received 0.15 mL of 10% AlCl_3_ (*w*/*v*) after 5 min. Then, 0.5 mL of 1M NaOH was added after 6 min. The addition of 0.5 mL of deionized water came next. The optical density was read at 510 nm using a Thermo Scientific Evolution 350 UV-Vis spectrophotometer (Waltham, MA, USA) after centrifugation at 8000× *g* for 4 min at room temperature (HERMEL -Z36HK— Wertheim, Germany) against the reagent blank. The total flavonoid content was expressed as mg of quercetin (Qur)/100 g sample (DW).

#### 2.8.3. Determination of Total Anthocyanin Concentration

Using the Abdel-Aal et al. [43] approach, total anthocyanins were extracted, and their amounts were determined. Samples (0.5 g) were extracted for 40 min at room temperature in 5 mL of acidified methanol (85% methanol and 15% 1.5 N HCl, *v*/*v*). The supernatant was transferred to a 10 mL volumetric flask after centrifugation at 20,000× *g* for 20 min at 4 °C (HERMEL -Z36HK— Wertheim, Germany), and the extraction was then carried out once more in the supernatant. The supernatants were combined. The absorbance of the mixture was read at 535 nm against a reagent blank. Kuromanin’s average molar extinction coefficient was used to compute the total anthocyanin concentration, which was then expressed as mg of kuromanin (KE)/100 g sample (DW). The concentration range (2.5–20.0 mg of KE) was used to create the calibration standard curve for kuromanin (Sigma–St. Louis, MO, USA), and the average molar extinction coefficient (Ɛ) was 25,700 ± 110 M^−1^ cm^−1^.

#### 2.8.4. Determination of Total Carotenoids and β- Carotene

The total carotenoids were extracted from the powdered samples of pigmented potatoes and their peels according to the previously reported method of Luterotti and Kljak, [44] with minor modifications. Total carotenoids were extracted from 25 g of powdered samples using a 75%(*v*/*v*) acetone/ethanol mixture. The total carotenoids (TC) were extracted from this mixture by n-hexane. TC was measured in the hexane extract spectrophotometrically at 452 nm by using n-hexane as a blank and using the specific absorptivity of 2500 dL g^−1^ cm^−1^, β- carotene was measured in the extract at 475 nm and calculated from the specific absorptivity of 2049 dLg^−1^ cm^−1^ according to Sharpless et al. [45].

### 2.9. Determination of DPPH and ABTS Radical Scavenging Capacity

The technique provided by Goffman and Bergman [33] was modified to determine the 2,2-diphenyl-1-picrylhydrazyl (DPPH) radical scavenging capacity. The reconstituted fraction was combined with 0.9 mL of DPPH solution (80 mg/L in 100% methanol) and incubated at room temperature for 30 min while kept out of the dark, and its absorbance was then read at 517 nm. Using the following formula, the percentages of DPPH radical scavenging activities in samples were calculated:

DPPH (%) activity = [(A0 − A1)/A0] × 100

where A0 is the absorbance of the blank and A1 is the absorbance of the sample.

The assay measures a substance’s capacity to scavenge a radical cation of (2, 2’- azino-bis ethylbenzthiazoline-6-sulfonic acid (ABTS) in relation to a reference butylated hydroxytoluene (BHT) according to the method of Re et al. [32]. The radical cation was made by combining 2.45 mM potassium persulfate (1/1, *v*/*v*) with 7 mM ABTS stock solution over a period of 4 to 16 h, depending on how quickly the reaction was finished and the absorbance stabilized. For measurements, ethanol was used to dilute the ABTS solution. After mixing 0.9 mL of ABTS+ with 0.1 mL of the test samples for 45 s, the photometric assay was performed, and measurements were made at 734 nm after one minute. The reduction in absorbance at various doses was used to calculate the antioxidant activity of the tested samples and standards (BHT and vitamin C) by using the following equation:

E= ((Ac − At)/Ac) × 100(1)

where At and Ac are, respectively, absorbance of tested samples or standard, and ABTS^.+^.

Results of the DPPH and ABTS assays were expressed as µmol Trolox equivalent antioxidant capacity per 100 g DW from the Trolox standard (0–25 μM). As described in our previous work [46] to standardize the DPPH and ABTS results, anti-radical efficiency (AE) and antioxidant activity index (AAI) were expressed according to the following:

AE = 1/(EC_50_ × TEc_50_) (2)

AAI = [Radical concentration in reaction mixture (μg/mL)/EC_50_ (μg/mL)]

### 2.10. UPLC-MS/MS Identification of the Individual Phenolic Compounds

The reconstituted acetonitrile extracts of phenolic compounds were analyzed to identify the individual total phenolic profile compounds as previously [21] described using a Waters Acquity UPLC–I class coupled with Xevo TQD MS (Milford, MA, U.S.A), Acquity UPLC BEH C18 1.7 µm–2.1 × 100 mm column flow rate 0.8 mL min^−1^, the injection volume 20 µL, Masslynix v4.1 software (Waters corporation, Milford, MA01757, USA)with a mass library, argon as collision cell gas inlet 7 psi, and nitrogen pressure 60 psi. The MS was set to an atmospheric pressure electrospray ionization (ESI) source, operated in negative ion mode. The electrospray capillary voltage was set to 3000 V, with a nebulizing gas flow rate of 12 L/h and a drying gas temperature of 300 °C. The mass spectrometry data was acquired in the scan mode (mass range *m*/*z* 100–1000). To scan the total phenolic profile, a binary gradient of (A) 0.5% formic acid in deionized water and (B) 100% methanol at 0.8 mL/min at 25 °C was used. The gradient used was: 0 min, 15% B; 0 to 15 min, linear gradient to 15% B; 15 to 25 min, linear gradient to 25% B; 25 to 35 min, linear gradient to 50% B; 35 to 50 min, linear gradient to 75% B; 50 to 55 min, linear return to 15% B; and 55 to 57 min, isocratic at 100% B to re-equilibrate. Mass spectral data were compared by the mass metabolite spectral library. The metabolite library uses high mass accuracy MS/MS spectra, R_t_ and isotopic information to identify and confirm compounds.

### 2.11. Statistical Analysis

The preparation of data for statistical analysis included testing for outliers, normalization, and z-score calculations where it was found to be normally distributed according to Mahmoud et al. [47,48]. A one-way ANOVA followed by Duncan’s test was performed to test the variations between samples based on the means ± standard deviations of three replicates for each of the variables. The principal component analysis (PCA) was performed using XLSTAT 2022^®^ (Addinsoft, Paris, France). Heatmap-based hierarchical clustering, followed by the similarity matrix for the Euclidian distances between the z-scores of the measured variables, was performed using the online platform “Morpheus Open“ (https://software.broadinstitute.org/morpheus; accessed on: 1 March 2023).

## 3. Results and Discussion

### 3.1. Proximate Analysis of Samples

The proximate chemical analysis of the raw materials SPF and SPPs is reported in Table 2. Generally, SPF has higher moisture, protein, total nitrogen, free amino acids, and total resistant starch. On the other hand, SPPs have higher fat, crude fiber, ash, carbohydrate, and starch. This is in line with the previous studies that concluded that the total starch, depending on the variety, is the highest component in SPs (between 50 and 80 g/100 g). Of these, resistant starch (RS) could be between 13.2 and 17.2%. On the other hand, protein was a minor fraction of the SPs (between 1.3 and ≈10 g/ 100 g) [49]. The SP protein has been linked with health benefits, including antioxidant activity, decreased levels of triglyceride in the blood, and anticancer and antiproliferative effects [50,51,52,53]. Furthermore, previous reports have indicated that the fat content in potato peels are two times higher than it is in peeled potatos [54,55,56].

**Figure 1:**
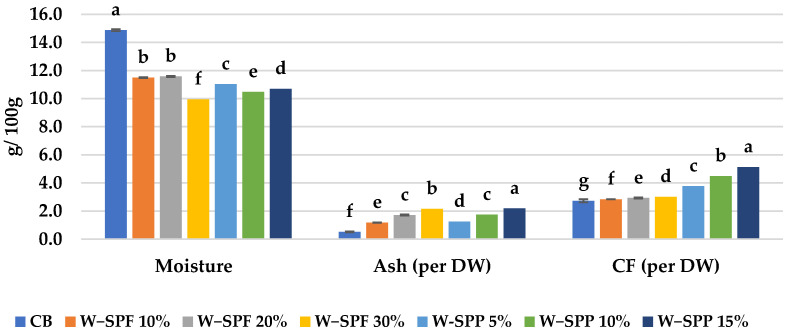
Proximate analysis of bread samples. CF: crude fiber, CB: control bread, W-: wheat flour, SPF: sweet potato flour, SPP: sweet potato peels. The letters a–g are indications of the significance within each variable according to Duncan’s test at *p* ≤ 0.05.

A proximate analysis of the control bread (CB) and the supplemented bread samples showed that CB had the highest moisture content among all samples (Figure 1). On the other hand, bread prepared by wheat-SPF or -SPP composites (W-SPF and W-SPP, respectively) at all levels was significantly higher in crude fiber and ash contents. However, the most predominant increase was in the sample W-SPP 15% (2.2 ash and 5.1 CF g/100 g). The same pattern was shown by Mau et al. [57] and Mitiku et al. [58] when they investigated the proximate composition of bread prepared from a wheat flour and wheat-SPF composite at 5, 10, and 15%. Furthermore, Kidane et al. [59] reported that both ash and fiber% increased in bread when fortified with orange-flesh SP flour at different mixing ratios (10, 20, and 30% SPF).

### 3.2. Mineral Composition of Samples

**Figure 2:**
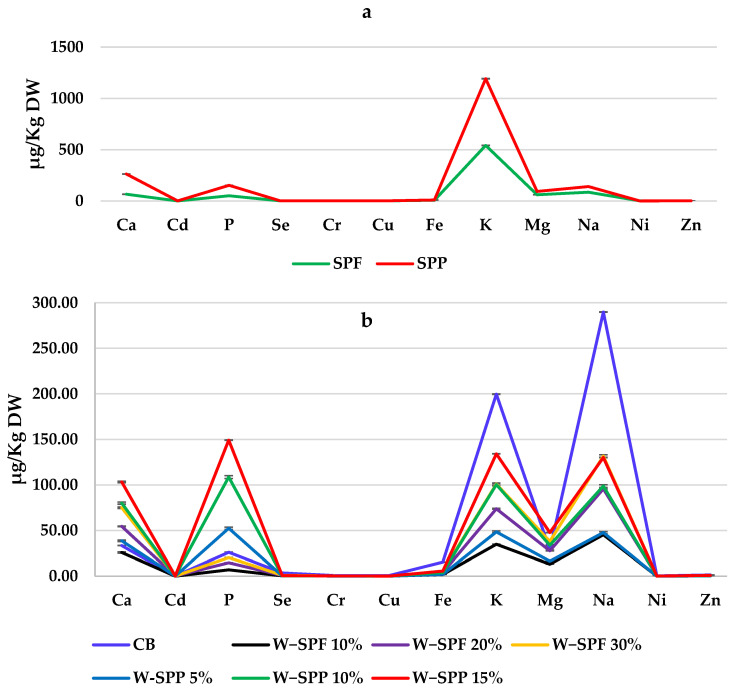
Elemental compositions of raw materials (**a**) and bread samples (**b**). CB: control bread, W−: wheat flour, SPF: sweet potato flour, SPP: sweet potato peel.

Twelve elements were identified and quantified by ICP-OES in the raw materials (SPPs and SPF) and bread samples. The mineral contents of SPPs were significantly higher than SPF except for the three elements Cr, Cu, and Ni (Figure 2a). Few studies have compared SPF and SPPs based on their elemental compositions. Among them are Salawu et al. [52] and Vaitkevičienė [57] who reported that SPPs have higher concentrations of Ca, Na, K, P, Mg, and Zn than SPF when investigating different SP cultivars.

As for the bread samples, no consensus pattern was observed when comparing the bread control with the substituted bread samples. However, samples of W-SPPs showed better mineral fortification ability compared with the W-SPF samples (Figure 2b). Previous reports showed inconsistencies in general when reporting on elemental concentrations in bakery products prepared by wheat-SPF or wheat-SPF-maize composites at different addition levels [58,60,61,62]. This might be attributed to the use of different identification methods and samples (peeled/unpeeled SPs).

Interestingly, CB was observed to have the highest K and P contents among all samples and this is in agreement with the previous report of Abo Raya et al. [63] who analyzed wheat flour with an extraction rate of 72% and stated the higher contents in potassium and phosphorus.

### 3.3. Total and Individual Phytochemicals

**Figure 3:**
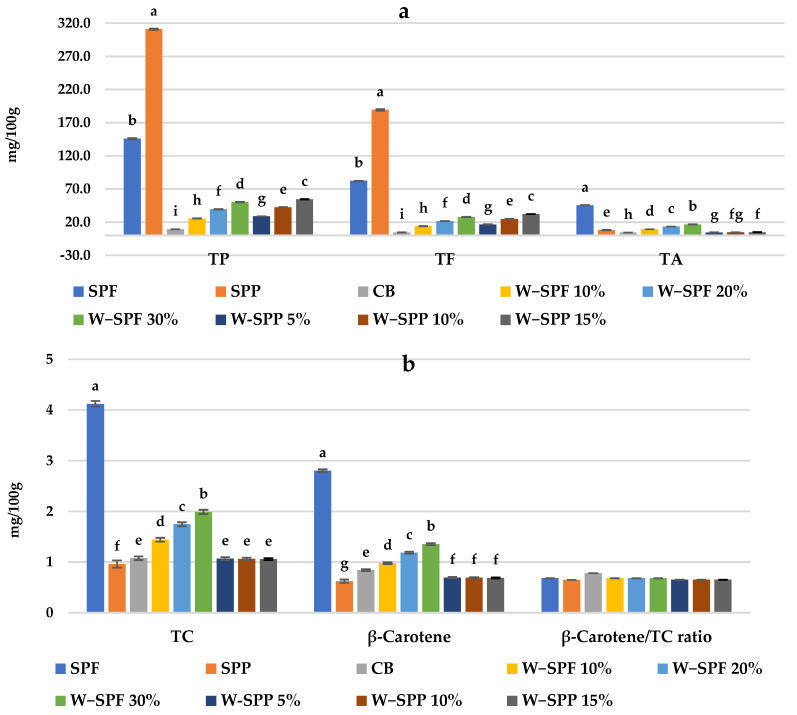
TP, TF, and TA (**a**) and TC, β-carotene, and their ratio (**b**) of raw materials and bread samples. TP: total phenol, TF: total flavonoids, TC: total carotenoids, TA: total anthocyanins, CB: control bread, W-: wheat flour, SPF: sweet potato flour, SPP: sweet potato peels. The letters a–i are indications of the significance within each variable according to Duncan’s test at *p* ≤ 0.05.

The phytochemical constituents of SPF and SPPs, and their bakery products are presented in Figure 3. Our results indicated that samples were different based on their total contents of phenolics (TPs), anthocyanins (TAs), flavonoids (TFs), and total carotenoids (TCs). Potato peels had the highest concentrations of TPs and TFs, while potato flour had the highest concentrations of TAs and TCs compared to the rest of the samples. Among bakery products, bread samples substituted with SPF or SPPs presented higher concentrations of TPs, TAs, TFs, and TCs compared to the control bread, and in correspondence to their addition levels. Samples fortified with SPPs gave higher ratings in TPs and TFs, while samples fortified with SPF gave higher ratings in TAs and TCs.

In this regard, Salawu et al. [55] indicated that potato peels have higher TP and TF contents compared to potato flesh, whereas Gabilondo et al. [64] indicated that potato flesh has higher TAs and TPs in its skin. We presume that the levels of these phytochemicals in sweet potato flesh versus peels are highly dependent on SP cultivars and cultivation conditions and methods. Both TAs and TCs are the main causes of flesh and peel colors [65]. Furthermore, β-carotene was the most abundant carotenoid with β-carotene/TC ratios between 78 and 65%. Similar results were reported in previous studies [16,17,54,66,67]. These compounds are responsible for giving the fortified bakery products a darker yellowish/orangish color compared to the control [68]. Our results indicated that the thermal treatment during packing has decreased TPs, TAs, TFs, and TCs in bread, which is in agreement with previous reports [69]. However, the fortified samples retained higher concentrations of these compounds compared to the control due to the protein- and starch-polyphenol interactions induced by the addition of SPF or SPPs, implying that they are still accessible for humans upon consumption [68,69,70]. For example, polyphenolic compounds, including anthocyanins and chlorogenic acids, were reported to inhibit the formation of carcinogens that are usually generated during the cooking and processing of foodstuff [49].

The individual phenolic contents of the SPF and SPPs’ ethanolic extracts have been characterized using UPLC-MS/MS (Table 3 and Figure S2). A total of 15 compounds were identified in SPPs and 13 compounds were detected in SPF. Most of these compounds belonged to the phenolic acids group (compounds 1–10). while five compounds (from 11 to 15) belonged to the flavonols group, which is in line with previous studies [1,54,69,71]. Furthermore, quantification of these fifteen compounds proved that their concentrations in SPPs are higher than SPF, except for gallic and caffeic acids. The most abundant compounds, in SPF and SPPs, respectively, were *p*-coumaric acid (6.6 and 12.97 µg/g), feruloyl-D-glucose (3.45 and 7.9 µg/g), eucomic acid (10.6 and 6.4 µg/g), gallic acid (1.53 and 7 µg/g), and ferulic acid (0.47 and 6.33 µg/g).

Zhu et al. [72] investigated the phenolic constituents in the methanolic extracts of fresh purple sweet potatoes (peels and flesh) using LC-MS. The authors focused on the determination of hydroxycinnamic acid derivatives and reported compounds, including mono- and di-caffeoylquinic acids and caffeic acid. Hydroxycinnamic acid derivatives are abundant in the cell walls of plant-based foods, so their concentrations are generally higher in their peels compared to their flesh [73]. Salawu et al. [55] have also studied the phenolic constituents of SP peels and flesh belonging to Nigerian cultivars, and they characterized compounds including gallic, caffeic, and chlorogenic acids, as well as rutin, quercetin, and kaempferol; the concentrations of these compounds were generally higher in the peel samples. As for anthocyanins, reports indicated that glucoside derivatives of ferulic acid were among the most abundant anthocyanins in different SP genotypes [16,74].

### 3.4. Antioxidant Activity

**Figure 4:**
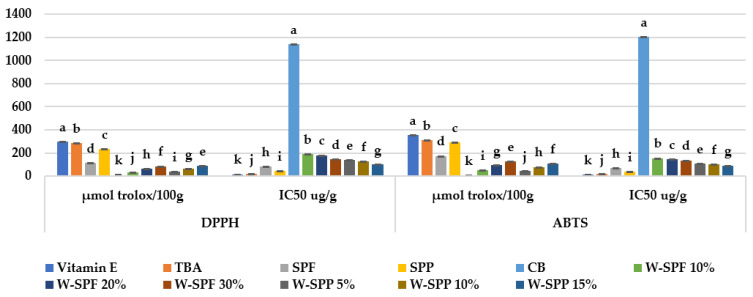
Antioxidant activity of samples against DPPH and ABTS compared to standard antioxidants expressed in μmol Trolox/100 g and the respective IC_50_. CB: control bread, W-: wheat flour, SPF: sweet potato flour, SPP: sweet potato peels. The letters a–k are indications of the significance within each variable according to Duncan’s test at *p* ≤ 0.05.

The Trolox equivalent antioxidant capacity (TEAC) of sample extracts was tested using the DPPH and ABTS assays (Figure 4a). Both raw materials presented significantly lower antioxidant capacities against DPPH and ABTS (113.35–173.76 for SPF and 235.2–291.73 μmol Trolox/100 g DW for SPP) when compared with TBA and vitamin E. These values were used to calculate the effective concentrations required to scavenge 50% of the reactive oxygen species, hence their IC_50_ values were higher than those of the standard antioxidants. In this regard, Teow et al. 2007 investigated the antioxidant capacities of sweet potato genotypes varying in flesh colors resulting in TEAC values (μmol Trolox/100 g DW) ranging between 16 and 14, and ~85 and 165 against DPPH and ABTS, respectively [75]. Additionally, Truong et al. 2007 compared the antioxidant capacities of peels and flesh of three American SP genotypes against DPPH; all peels samples presented better antioxidant activity (470–710 μmol Trolox/100 g DW) compared to flesh samples (150–200 μmol Trolox/100 g DW) [76]. Comparable results were reported by Cui and Zhu [16] when they assessed the TEAC values of seven SP flours made from New Zealand SP cultivars, where the mean values were 270 and 339 μmol Trolox/100 g DW against ABTS and DPPH, respectively.

As for bread samples, control bread has the lowest antioxidant activity among all samples, with values equal to 9.89 and 7.525 μmol Trolox/100 g against DPPH and ABTS, respectively (Figure 4). Fortification with SPF or SPPs at all levels has significantly increased their antioxidant activities. Thereby, a positive trend exists between the addition levels and the TEAC values of the respective samples. Interestingly, W-SPF samples showed better values when tested against ABTS, while W-SPP samples presented better values when tested against DPPH. When compared with the raw materials in their respective fortified breads, a decline in antioxidant activity could be observed. Previous studies attributed that to the lowering of the polyphenolic contents because (I) the lower extractability resulted from protein—or starch-polyphenol interactions, (II) enzymatic oxidation during dough mixing while administrating water and, unintentionally, O_2_, and (III) heat treatment during baking [69,77,78,79].

Other studies focused on purple SP root varieties because they exhibit the highest concentration of polyphenols, thus higher antioxidant activity compared to white, yellow, or orange SP cultivars [64,67,80]. However, preparing doughs for bread with such SP products results in the weakening of the doughs and has a technological limitation due to the purple color of the roots. These results are in line with the results of Han and Koh [78] and Xu et al. [79] who fortified doughs with different phenolic compounds aiming to improve their antioxidant activity and adversely made doughs with lower stability compared to the control. It is known that doughs with lower stabilities have limited bakery applications [81], thus these SP cultivars are more suitable for other ways of consumption, e.g., steaming, baking, boiling, and mashing.

We further investigate the correlation between TEAC values and the total contents of TPs, TAs, TFs, and TCs, in addition to β-carotene as the major TC constituent, using the Pearson correlation matrix (Table 4). The results showed a positive correlation between DPPH and ABTS, and the TEAC values for both TPs and TFs, implying that they are the major constituents that contributed to the total antioxidant activity of the samples. Comparably, previous reports suggested that crude extracts of TP contents from dietary plants could significantly induce their antioxidant capacities [49,60].

**Figure 5:**
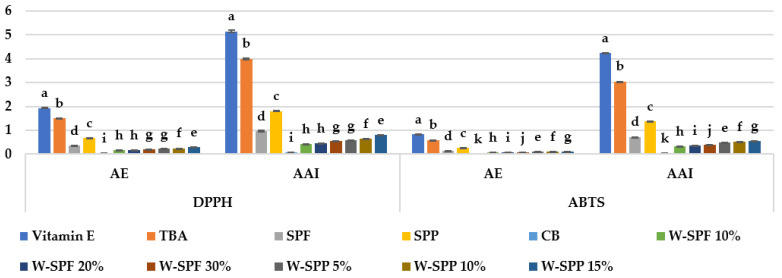
Antiradical efficiency (AE), and antioxidant activity index (AAI) of samples against DPPH and ABTS compared to standard antioxidants. CB: control bread, W-: wheat flour, SPF: sweet potato flour, SPP: sweet potato peels. The letters a–k are indications of the significance within each variable according to Duncan’s test at *p* ≤ 0.05.

Both the antioxidant activity index (AAI) and antiradical efficiency (AE) were proposed to cope with the lack of standardization for the DPPH method, thus the difficulty when comparing the antioxidant activities of different plant extracts or pure standard compounds [34,35]. We calculated both parameters to facilitate future comparisons between the current report and future studies. Theoretically, there are no limitations to calculating the AAI and AE for other antiradical species with similar mechanisms to DPPH, i.e., ABTS. However, we could not find such reports, therefore, we calculated both parameters for the ABTS assay, in addition to DPPH, and the results are presented in Figure 5.

To test the accountability of introducing AAI and AE to the ABTS assay results, a Pearson correlation matrix was generated for the TEAC values expressed in μmol Trolox/100 g, IC_50_, AE, and AAI for the standard antioxidants vitamin (E) and TBA, and the extracts of sweet potato (peels and pulp) and bread samples (control and fortified; Table 5). According to the Pearson correlation, the values AE and AAI for ABTS correlated significantly with those of DPPH (*p* < 0.0001; R^2^ of 89 and 99%). Thus, we conclude that AE and AAI could be calculated for ABTS and would recommend using it for other plant matrixes as well as other standard antioxidants.

### 3.5. Dough Mixing Properties

The rheological properties of wheat, wheat-SPF, and wheat-SPP doughs were studied using the Farinograph test (Table 6 and Figure S1). Our results indicated that doughs prepared from wheat-SPF and wheat-SPP composites have significantly higher water absorption rates (WA%) compared to the control. This might be attributed to the chemical characteristics of tubular crops, i.e., they contain higher concentrations of polyphenols with their ability to form water-hydrogen bonds as well as the higher swelling power of their starches [3,30,82,83]. The higher fiber contents contribute as well to increasing the WA% due to their high content of hydroxyl groups, which facilitate their ability to retain water molecules due to hydrogen bonding [84]. Similarly, an increase in the dough development time (DTT) was observed with the addition of SPF or SPPs from 1.3 min in the CB sample to 1.3–5.2 and 6.4–7.1 min in wheat-SPF and wheat-SPP composites, respectively due to the same reasons that cause the increase in WA%, which is supported by previous studies [84,85].

Furthermore, an inverse relationship was recorded between dough stability and the additional levels of wheat-SPF or wheat-SPPs, thus the highest stability was noted at their lowest addition levels. However, all observations were higher than the control. On the other hand, increasing the substitution levels of SPF or SPPs increased the mixing tolerance index (MTI) of the doughs, thus reducing their time to break down. The increase in dough stability and the decrease in MTI are indications of strength improvements for our doughs, thereby increasing their usage in different bakery applications [30]. An overall evaluation of the rheological properties of our composites could be summarized by the Farinograph quality number [86], and thus it was clear that five treatments resulted in improved doughs compared to the control. As for the sample W-SPF 10%, it has an insignificant difference from the control.

Wheat flour glutens, upon hydration and mixing, can form a network that determines the viscoelasticity property and quality of the formed dough [87]. This network is affected by gluten concentrations in wheat flour and could be strengthened by the presence of Ca^2+^ and maintained by polyphenols and other antioxidants [30,88,89]. These antioxidants protect the intermolecular S-S bonds of the formed networks against oxidation [30,90,91]. Our results indicated increases in Ca^2+^ and polyphenols compared to the control. This might be the reason for the improvement in dough quality after fortification. However, a further increase in polyphenol contents brings adverse effects on the quality of the dough by decreasing the number of disulfide bonds and thus decreasing dough stability [91].

### 3.6. Color

**Figure 6:**
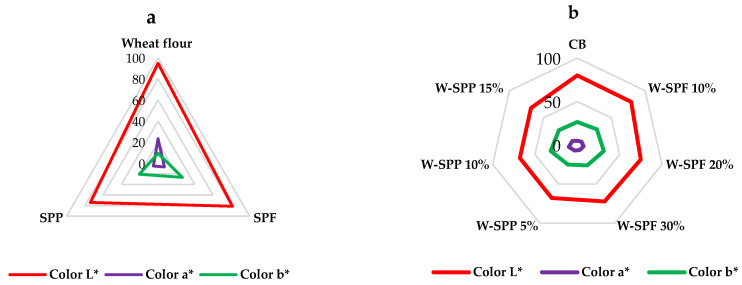
Color parameters of raw materials (**a**) and bread samples (**b**). CB: control bread, W-: wheat flour, SPF: sweet potato flour, SPP: sweet potato peels.

Our results indicated that the color of the raw materials ranged in brightness depending on the type of sample, with wheat flour having the highest lightness followed by the SPF and then the SPPs (Figure 6 and Table S1). These parameters are in line with previous studies, which indicated that the color variation might be attributed to the difference in their contents due to proteins, fibers, elements, phytochemicals, and other impurities [3,16]. Regarding the bakery products, all samples showed a significant decrease in brightness (L*) compared to the control bread (Figure 6 and Table S1), except W-SPF 10%. On the other hand, all wheat-SP composites showed a significant increase in redness (a*). As for yellowness (b*), the highest addition in the two composite formulations (W-SPF 30% and W-SPP 15%) did not show any significant differences from the CB sample, while the W-SPP 5% was significantly lower than it. The rest of the wheat-SP composite formulation showed a significant increase in redness when compared with the control.

Previous researchers have explored the changes in color parameters of different bakery products influenced by the addition of peeled and unpeeled sweet potato flour to wheat flour. In this regard, Chikpah et al. [92] investigated the qualities and characteristics of bread fortified with peeled or unpeeled orange-fleshed SP flour. Generally, with the addition of peeled or unpeeled SP flour, the brightness values decreased whereas the redness and yellowness increased. However, samples prepared from the unpeeled SP flour showed lower L* and higher a* and b* values than those prepared with peeled SP flour. Chikpah et al. [92] summarized that the color changes are attributed to the differences in baking time and temperature, addition ratios, and the concentrations of phytochemicals like anthocyanins and β-carotenes in the SPF samples. Furthermore, increasing the addition ratios of SPF to wheat flour does not always increase the a* and b* compared to the control samples as reported by Aritonang et al. [23] and Singh et al. [93]. Where Singh et al. [93] stated cookies prepared with 100% wheat flour or wheat-SPF 80:20% showed no significant differences in redness or yellowness. Furthermore, Aritonang et al. [23] reported that the increase in the addition ratio from 80:20% to 60:40% wheat-SPF had no significant effect on the redness of the sweet bun.

### 3.7. Sensory Analysis

**Figure 7:**
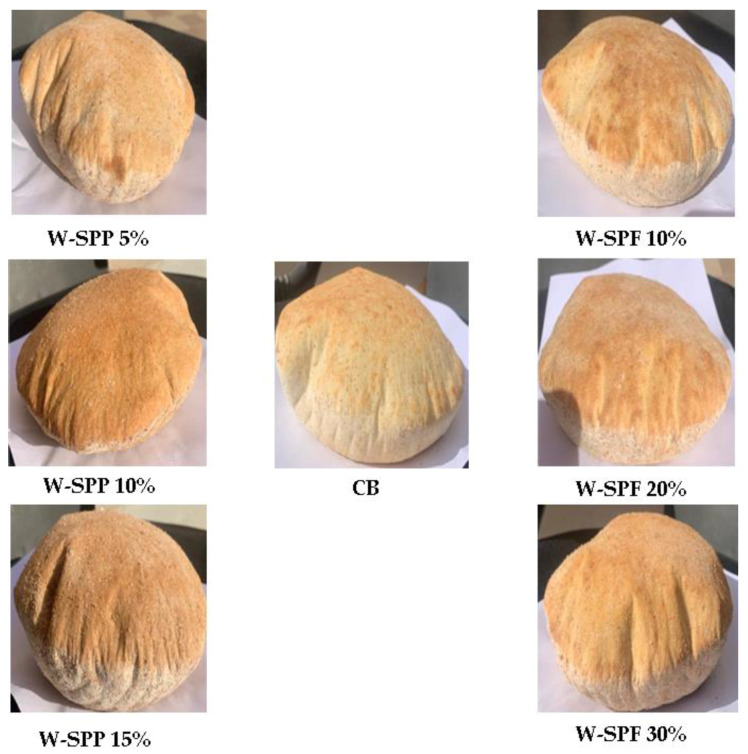
Baked control and fortified bread samples. Samples are puffed and have two separate layers. CB: control bread, W−: wheat flour, SPF: sweet potato flour, SPP: sweet potato peel.

**Figure 8:**
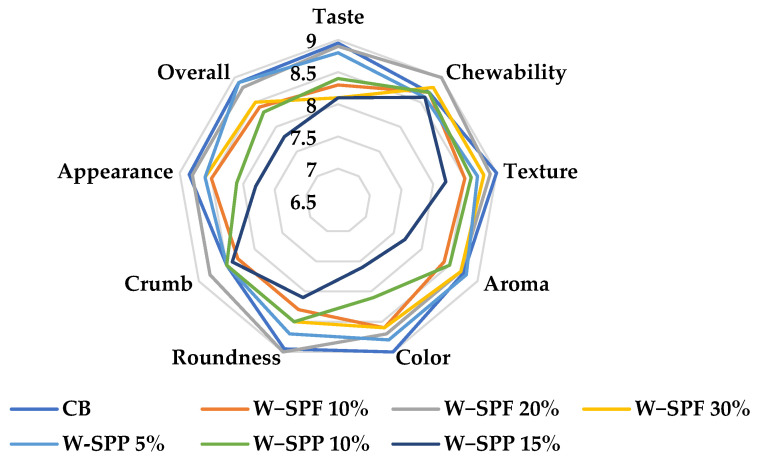
Sensory evaluation of baked control and fortified bread samples. CB: control bread, W−: wheat flour, SPF: sweet potato flour, SPP: sweet potato peel.

Control and supplemented bread samples (Figure 7) have been evaluated for their appearance, color, crumbliness, roundness, texture, chewability, taste, aroma, and overall impression (Figure 8 and Table S2). Generally, all samples were insignificantly lower than the control based on the rates of the nine attributes. Only the sample W-SPPs 15% was significantly different in color from the control. This indicates that fortification of bread with SPF or SPPs has maintained the sensory quality of the supplemented bread while improving the rheological and nutritional values compared to the control. In detail, it was found that the sample W-SPPs 15% possessed the lowest ratings of the attributes chewability, color, roundness, appearance, texture, aroma, and overall evaluation. Under the same preparation and baking conditions, the change in color is closely related to the overall contents of the product, especially from elements, pigmented phytochemicals, and precursors of the Maillard reaction [94]. Furthermore, the increase in fiber and polyphenols could affect the elasticity of dough, thus the texture, roundness, and chewability of the final product [84,95]. These factors combined might contribute to the overall evaluation of the bread.

On the other hand, the only samples that recorded increases in their ratings compared to the control bread were W-SPF 20% in the attributes chewability, roundness, and crumbliness, while W-SPF 30% recorded an increase in the chewability, and finally W-SPPs 5% presented an increase in the aroma compared to the control bread.

Our results are in agreement with the report of Idolo [60] who compared bread samples prepared from wheat flour and wheat:SPF composites (85:15, 80:20, and 75:25) based on their color, texture, aroma, taste, and overall acceptability. It was noted that all fortified samples were insignificantly different from the control based on the examined sensory attributes, except for the wheat: SPF at 75:25 was significantly different in color from the control. Mitiku et al. [58] evaluated bread prepared from partially substituted wheat flour SPF (100:0, 95:5, 90:10, 85:15, 80:20, and 75:25) based on their color, texture, taste, and overall acceptability. It was reported that the increase in the SPF addition ratio caused a decrease in the rates of the sensory attributes. However, these differences were insignificant at the lower substitution levels (95:5 and 90:10) [58]. Furthermore, the scores of the rest of the samples were never less than 6 points on the hedonic scale, indicating their general acceptability, which is in line with our results.

### 3.8. Overall Evaluation of Samples Based on Chemometric Methods

**Figure 9:**
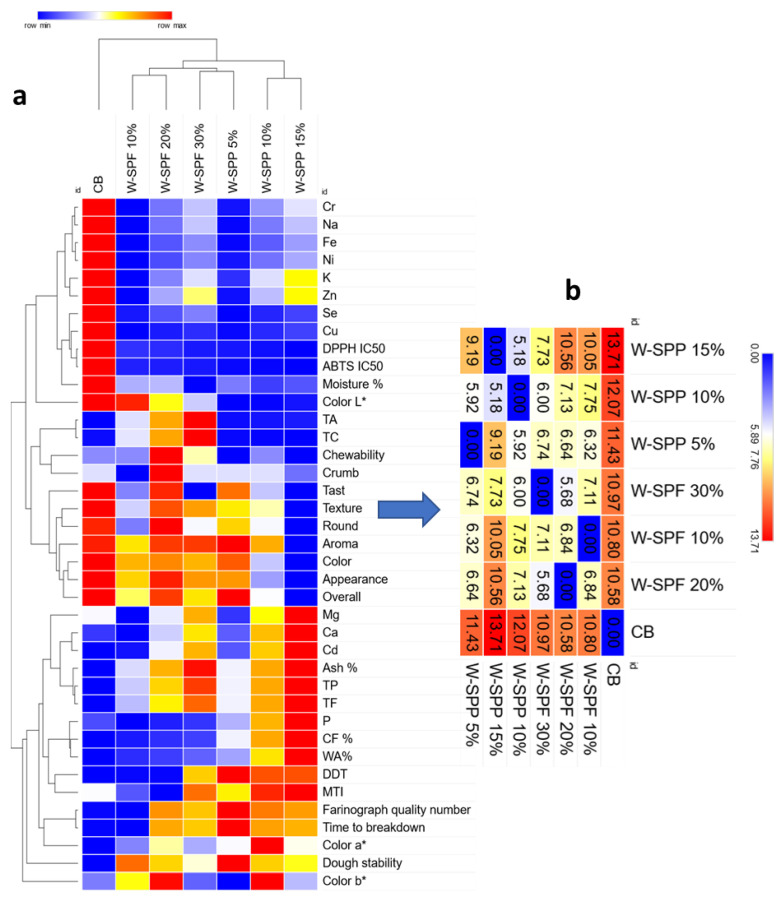
Heatmap−based clustering of samples and their variables based on Euclidean distances between the z-−score values (**a**) followed by a similarity matrix that ranks the sample to the control (**b**).

Generally, the z-score values of the measured physical, chemical, and technological variables were used to perform chemometric evaluations of the data. First, we performed a hierarchical-based heatmap clustering to provide an assessment of the similarities and variations among bread samples (Figure 9a). Hence, samples were clustered into three groups in which the control bread was in group C1, all W-SPF samples + W-SPPs at 5% in group C2, and finally, W-SPPs at 10 and 15% were in group C3. On the other hand, the variables clustered into two major groups and separated the samples as follows: (I) The control bread was separated from the rest of the samples due to its high concentration of seven elements (Cr, Na, Fe, Ni, Zn, Se, and Cu) and moisture%. On the other hand, CB presented the lowest values of antioxidant activity, ash%, TPs, TFs, and dough stability, (II) the sample W-SPF 20% presented the highest chewability and crumbliness sensor evaluations, (III), the sample W-SPF 30% showed the highest TA and TC contents, (IV), the sample W-SPP 5% had the highest dough development time, time to breakdown, dough stability, and Farinograph quality number while having the lowest color b* value, (V), the sample W-SPPs 10% had the highest color b* value, and finally, (VI) the sample W-SPPs 15% had the lowest sensory evaluation parameters of texture, round, aroma, color, appearance, and overall acceptability, while retaining the highest Mg, Ca, Cd, TP, TF, CF%, and WA%. However, to have a deeper insight into these differences and their significance, it is essential to cross-check the previous sections of the manuscript in the respective part of each analysis.

We further performed a similarity matrix based on the Euclidean distances between the clustered variables to rank the samples based on their similarity to the control bread (Figure 9b). It was revealed that sample W-SPF 20% has the lowest variation from the control (10.8%), while sample W-SPPs 15% is the farthest from the control (13.71%). Based on that, the fortified samples are closer to each other than the control.

**Figure 10:**
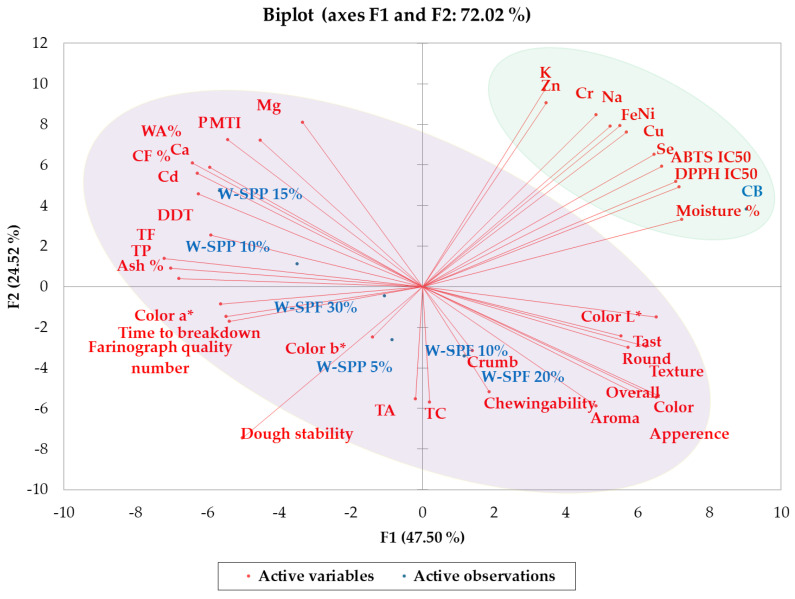
PCA-−bi Plot of bread samples. CB: control bread, W−: wheat flour, SPF: sweet potato flour, SPP: sweet potato peel.

The principal component analysis (PCA) confirmed these results when exploring the correlation between variables and their respective samples (Figure 10). Thereby, the PCA-bi plot could explain 77% of the variation between bread samples, and two groups were observed on the PCA-bi plot. The first group contained all the fortified samples and implied their association with the sensory attributes, physical properties, phytochemical compositions, and higher antioxidant activities. The second group contains the control and implies a generally poorer nutritional value, except for the elemental composition.

## 4. Conclusions

The target of our work was to develop nutritious Arabic bread products prepared from wheat-sweet potato peels or flour composites. To this aim, different addition levels were used, and the dough- and bread-making processes were optimized and validated using chemometric methods. It was revealed that bread prepared from either wheat-sweet potato peels or flour composites has higher elemental concentrations, bioactive molecules, and antioxidant capacity compared to the control. Furthermore, these blends produced dough mixes with improved rheological properties compared to the control, and the final bread products possessed comparable sensory qualities to the control. We assume the increase in calcium ions and polyphenols was responsible for maintaining and protecting the gluten network of the dough. However, the further increase in the addition ratios of SPF or SPPs caused adverse effects on the dough characteristics, thus sensory acceptability of the final products.

We further examined the possibility of measuring the AAI and AE values for ABTS, comparably with DPPH. Accordingly, the Pearson correlation matrix showed a significant correlation between AE and AAI for ABTS with TEAC values of DPPH (*p* < 0.0001; R2 of 89 and 99%). Thereby, we recommend using this method to overcome the lack of standardization when reading DPPH and ABTS methods, e.g., measuring the absorbance at different times and using different concentrations of the reagents.

## Figures and Tables

**Table 1:** Mixing ratios of control and substituted bread samples and their labels.

Ingredients	Bread Samples						
	CB	W-SPF 10%	W-SPF 20%	W-SPF 30%	W-SPP 5%	W-SPP 10%	W-SPP 15%
Wheat flour 72%(g)	100	90	80	70	95	90	85
SPF (g)	0	10	20	30	0	0	0
SPP (g)	0	0	0	0	5	10	15
Sugar (g)	2	2	2	2	2	2	2
Salt (g)	2	2	2	2	2	2	2
Water (mL)	60	60.5	60.9	61.5	61.3	63	64.3
Yeast (g)	1.5	1.5	1.5	1.5	1.5	1.5	1.5

CF: crude fiber, CB: control bread, W-: wheat flour, SPF: sweet potato flour, SPP: sweet potato peel.

**Table 2:** Proximate analysis of sweet potato flour (SPF) sweet potato peel (SPP).

Parameters (g/100 g)		SPF	SPP
Moisture		38.4 ± 0.2 ^a^	4.9 ± 0.1 ^b^
**per dry weight (DW)**	Fat	0.9 ± 0.0 ^b^	3.2 ± 0.1 ^a^
	Protein	10.1 ± 0.1 ^a^	8.3 ± 0.0 ^b^
	Total Nitrogen	1.6 ± 0.0 ^a^	1.3 ± 0.0 ^b^
	Ash	5.9 ± 0.1 ^b^	11.6 ± 0.2 ^a^
	Carbohydrate	66.1 ± 0.0 ^b^	70.9 ± 0.4 ^a^
	Starch	48.8 ± 0.3 ^b^	57.6 ± 0.4 ^a^
	Total Resistant Starch	17.3 ± 0.3 ^a^	13.3 ± 0.8 ^b^
	Crude Fiber	3.6 ± 0.1 ^b^	18.7 ± 0.1 ^a^
	Free Amino Acids (as lysine)	4.1 ± 0.0 ^a^	1.8 ± 0.1 ^b^

Data presented as the mean ± SD of three replicates. The letters a, b are indications of the significant within each raw according to Duncan’s test at *p* ≤ 0.05.

**Table 3:** LC-Tandem MS identification of phenolic compounds content in sweet potato four (SPF) and sweet potato four (SPP) extracts.

Compound Name		Ret. Time [min]	[M + H]^−^	Base Beak *m*/*z*	Amount µg/g	
					SPF	SPP
1	*p*-Coumaric acid	1.974	163	93	6.60	12.97
2	Gallic acid	2.090	169	125	10.60	6.40
3	Ferulic acid	2.230	193	134	0.47	6.33
4	Caffeic acid	3.926	197	135	0.83	0.76
5	Eucomic acid	5.278	239	179	1.53	7.00
6	Feruloyl-D-glucose	7.664	355	193	3.45	7.90
7	Chlorogenic acid	10.606	353	353	ND	1.11
8	Caffeoylquinic acid	17.727	353	353	0.51	1.66
9	Catechin	18.155	289	289	0.75	2.49
10	Kaempferol	21.337	285	185	ND	2.68
11	Quercetin-3-O-glucoside	21.493	463	463	0.76	1.56
12	Quercetin-3-O-galactoside	21.808	463	301	0.23	0.92
13	Quercetin diglucoside	29.508	625	463	0.44	0.59
14	1,3-Dicaffeoylquinic acid	31.302	515	353	0.32	0.42
15	1,5-Dicaffeoylquinic acid	31.489	515	353	0.48	0.64

**Table 4:** Pearson correlation between antioxidant capacity essays and the phytochemical fractions.

Antioxidant Capacity Essay ^a^	TP	TF	TA	TC	β-Carotene
DPPH	**0.917**	**0.919**	0.167	0.050	0.021
ABTS	**0.901**	**0.898**	0.293	0.182	0.153

^a^ expressed in μmol Trolox/100 g. Numbers in bold imply that their values are significant (*p* < 0.0001).

**Table 5:** Pearson correlation between antioxidant capacity essays DPPH and ABTS.

Antioxidant Capacity Essay		DPPH				ABTS			
		μmol Trolox/100 g	IC_50_ μg/g	AE	AAI	μmol Trolox/100 g	IC_50_ μg/g	AE	AAI
DPPH	μmol Trolox/100 g	**1**	**−0.507**	**0.990**	**−0.471**	**0.934**	**0.934**	**0.920**	**0.920**
	IC_50_ μg/g	**−0.507**	**1**	**−0.548**	**0.999**	−0.416	−0.416	−0.408	−0.408
	AE	**0.990**	**−0.548**	**1**	**−0.513**	**0.902**	**0.902**	**0.889**	**0.889**
	AAI	**−0.471**	**0.999**	**−0.513**	**1**	−0.384	−0.384	−0.377	−0.377
ABTS	μmol Trolox/100 g	**0.934**	−0.416	**0.902**	−0.384	**1**	**1.000**	**0.998**	**0.998**
	IC_50_ μg/g	**0.934**	−0.416	**0.902**	−0.384	**1.000**	**1**	**0.998**	**0.998**
	AE	**0.920**	−0.408	**0.889**	−0.377	**0.998**	**0.998**	**1**	**1.000**
	AAI	**0.920**	−0.408	**0.889**	−0.377	**0.998**	**0.998**	**1.000**	**1**

Numbers in bold imply that their values are significant (*p* < 0.0001).

**Table 6:** Farinograph parameters of control bread and substituted with different levels of sweet potato four (SPF) or peel (SPP).

Sample		WA%	DDT	Dough stability	MTI	Time to Breakdown	Farinograph Quality Number
CB		60.0 ± 0.1 ^f^	1.3 ± 0.0 ^d^	3.5 ± 0.1 ^f^	48.7 ± 3.1 ^e^	3.5 ± 0.0 ^d^	39.0 ± 1.0 ^d^
W-SPF %	10	60.4 ± 0.1 ^e^	1.4 ± 0.0 ^d^	8.2 ± 0.0 ^b^	23.3 ± 0.6 ^f^	3.5 ± 0.0 ^d^	38.3 ± 0.6 ^d^
	20	60.5 ± 0.1 ^e^	1.3 ± 0.2 ^d^	7.3 ± 0.0 ^c^	10.3 ± 0.6 ^g^	8.4 ± 0.1 ^b^	86.0 ± 1.0 ^bc^
	30	60.8 ± 0.1 ^d^	5.2 ± 0.0 ^c^	6.2 ± 0.0 ^e^	80.3 ± 0.6 ^c^	8.1 ± 0.0 ^c^	81.0 ± 1.0 ^c^
W-SPP %	5	61.3 ± 0.1 ^c^	7.1 ± 0.0 ^a^	9.2 ± 0.2 ^a^	62.7 ± 2.5 ^d^	10.2 ± 0.3 ^a^	100.3 ± 6.8 ^a^
	10	62.7 ± 0.3 ^b^	6.4 ± 0.1 ^b^	7.3 ± 0.0 ^c^	90.0 ± 2.0 ^b^	8.5 ± 0.0 ^b^	88.3 ± 0.6 ^b^
	15	64.3 ± 0.2 ^a^	6.4 ± 0.1 ^b^	6.8 ± 0.3 ^d^	95.0 ± 2.0 ^a^	8.3 ± 0.1 ^b^	85.0 ± 2.0 ^bc^

Data presented as the mean ± SD of three replicates. The letters a–g are indications of the significance within each variable according to Duncan’s test at *p* ≤ 0.05. WA%: water absorption rates, DTT: dough development time, MTI: the mixing tolerance index.

## Data Availability

All data are available within the manuscript.

## Supplementary Materials

The following supporting information can be downloaded at: https://www.mdpi.com/article/10.3390/foods12081658/s1, Figure S1: Farinograph diagrams of samples; Figure S2. LC-Tandem MS chromatograms of sweet potato peel (SPP; a) and sweet potato flour (SPF; b); Table S1: Color evaluation of bread containing substituted flour with different levels of SPF or SPP; Table S2: Sensory evaluation of bread containing substituted flour with different levels of SPF or SPP.
