# Supplementary material for: The Chemical, Rheological, and Sensorial Characteristics of Arabic Bread Prepared from Wheat-Orange Sweet Potatoes Flour or Peel
Source: Foods. 2023 Apr 15;12(8):1658. doi: 10.3390/foods12081658 (PMC10137974; doi:10.3390/foods12081658)
Supplement: Supplementary file 1 [file foods-2304801-supplementary.pdf › foods-2304801-supplementary.pdf]

Tables

Table S1. Color evaluation of bread containing substituted flour with different levels of sweet potato four (SPF) and sweet potato four (SPP)

**Sample**		**Color L***	**Color a***	**Color b***
**CB**		80.1±0.3^{a}	4.8±0.1^{d}	26.5±0.3^{c}
**W-SPF %**	**10**	79.4±0.9^{a}	6.01±0.6^{c}	28.8±0.9^{b}
	**20**	75±0.3^{b}	7.5±0.1^{b}	31.3±0.3^{a}
	**30**	72.3±0.4^{c}	6.4±0.1^{c}	26.2±0.3^{c}
**W-SPP %**	**5**	67.8±0.5^{d}	7.2±0.4^{b}	25.1±0.2^{d}
	**10**	67.9±0.9^{d}	10.1±0.1^{a}	31.4±1.1^{a}
	**15**	68.2±1.0^{d}	7.3±0.2^{b}	27.1±0.3^{c}

Each value represents the mean (±SD) of three different replications. The different letters on the same column show a significant difference according to Duncan's test at $p \leq 0.05$.

Table S2. Sensory evaluation of bread containing substituted flour with different levels of sweet potato four (SPF) and sweet potato four (SPP)

Sample		Appearance	Color	Crumb	Roundness	Texture	Chewability	Taste	Aroma	Overall
**CB**		8.9±1.0[a]	9.0±0.9[a]	8.5±0.9[a]	9.0±0.8[a]	9.0±1.0[a]	8.7±0.9[a]	9.0±0.9[a]	8.8±1[a]	8.9±0.8[a]
**B-SPF %**	**10**	8.5±1.5[a]	8.6±1.6[ab]	8.3±1.3[a]	8.3±1.5[a]	8.5±1.5[a]	8.7±1.5[a]	8.3±1.3[a]	8.4±1[a]	8.4±1.5[a]
	**20**	8.8±1.2[a]	8.7±1.3[a]	8.8±1.4[a]	9.0±1.3[a]	8.9±1.4[a]	9.0±1.2[a]	8.9±1.0[a]	8.7±1[a]	8.8±1.2[a]
	**30**	8.6±1.0[a]	8.6±1.1[ab]	8.5±1.3[a]	8.5±1.1[a]	8.8±1.1[a]	8.8±0.9[a]	8.1±1.1[a]	8.7±2[a]	8.5±0.8[a]
**B-SPP %**	**5**	8.6±1.6[a]	8.8±0.8[a]	8.5±1.3[a]	8.7±1.6[a]	8.7±0.7[a]	8.6±0.7[a]	8.8±0.6[a]	8.8±1[a]	8.9±0.9[a]
	**10**	8.1±1.4[a]	8.1±0.9[ab]	8.5±1.1[a]	8.5±1.6[a]	8.6±1.2[a]	8.7±1.1[a]	8.4±1.7[a]	8.5±1[a]	8.3±1.6[a]
	**15**	7.8±1.1[a]	7.6±1.1[b]	8.4±1.3[a]	8.1±1.4[a]	8.2±1.2[a]	8.6±1.1[a]	8.1±1.4[a]	7.7±2[a]	7.8±1.0[a]

Each value represents the mean (±SD) of three different replications. The different letters on the same column show a significant difference according to Duncan's test at $p \leq 0.05$.

Figures

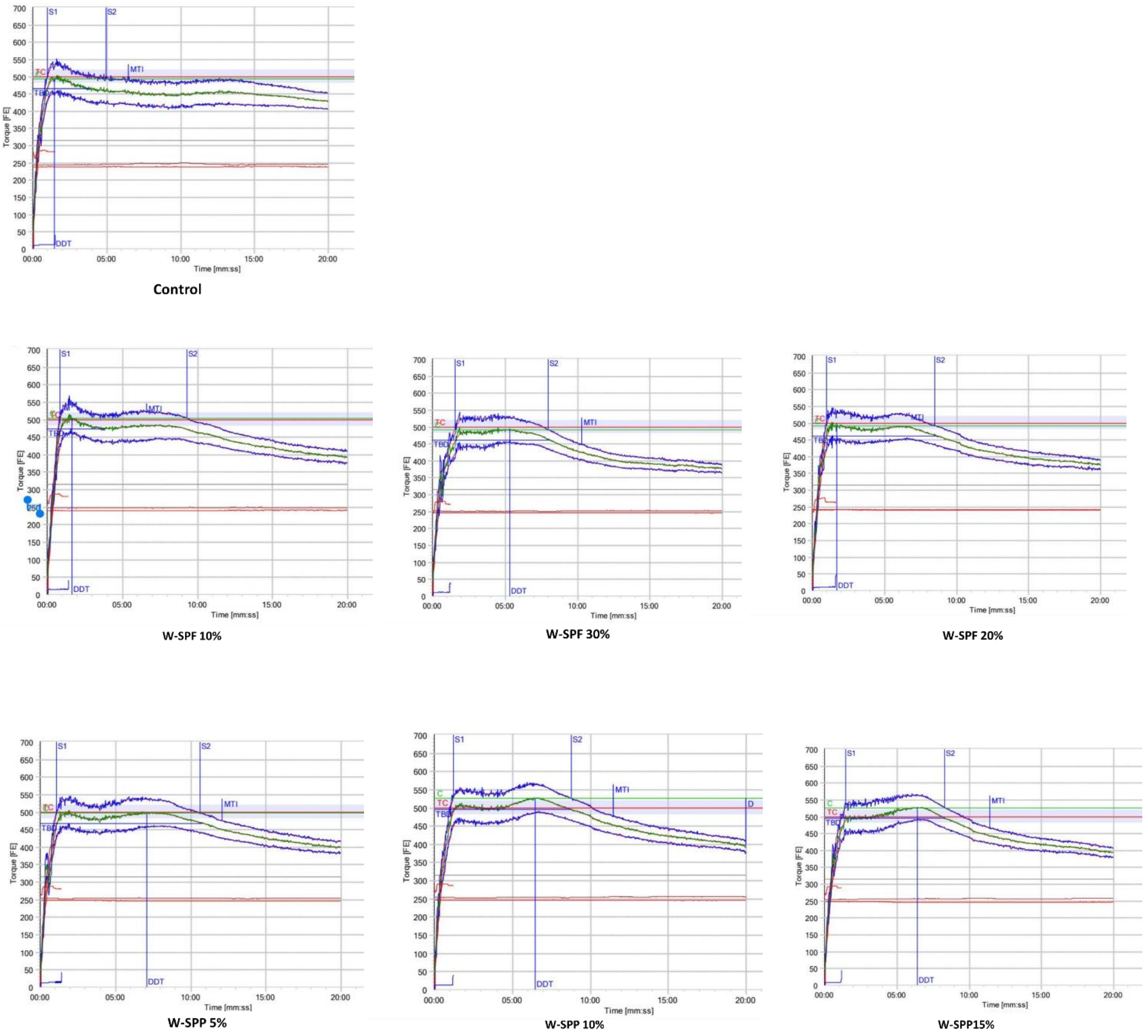

Figure S1. Farinograph diagrams of samples. CB: control bread, W-: wheat flour, SPF: sweet potato flour, SPP: sweet potato peel.

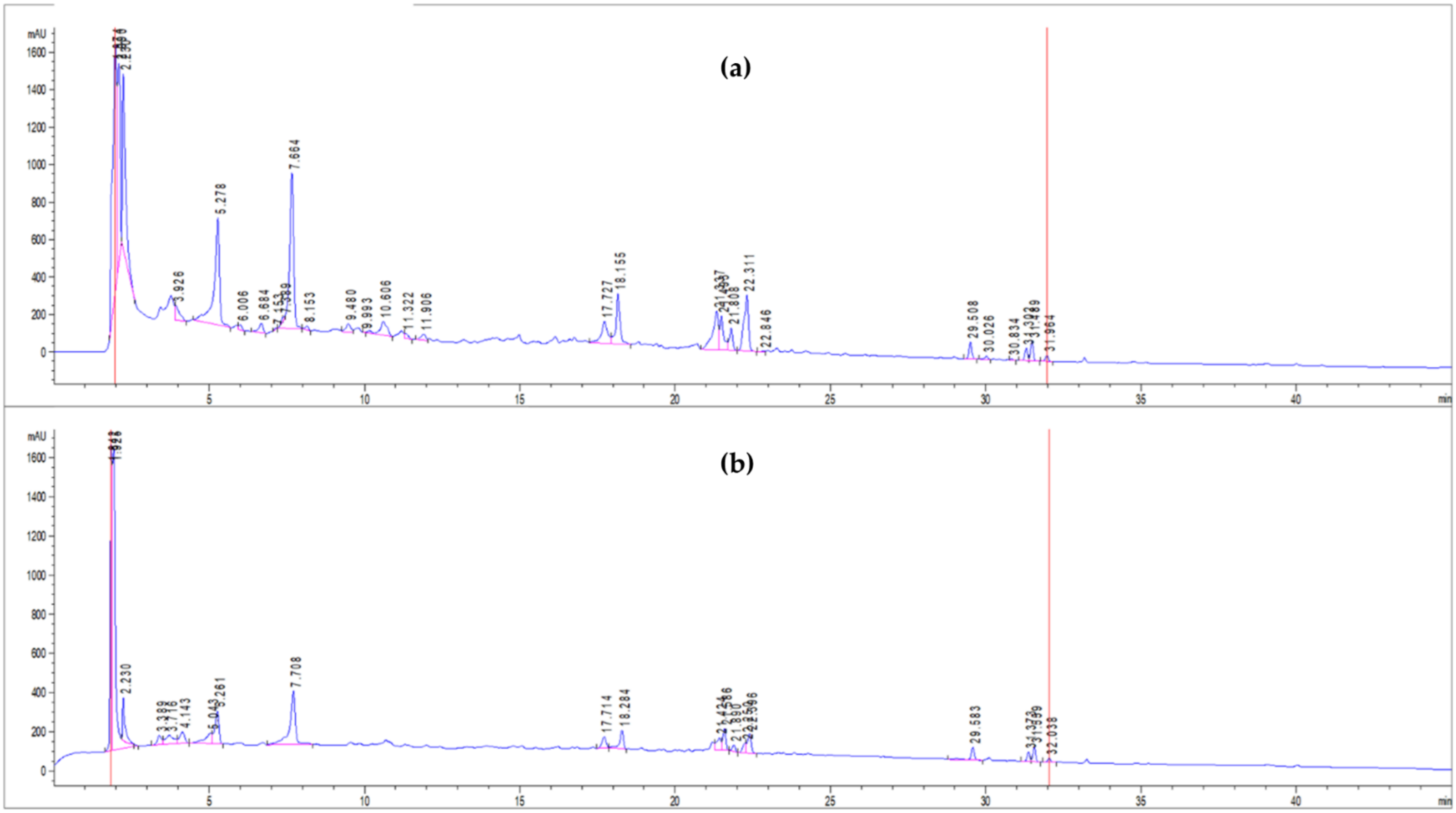

Figure S2. LC-Tandem MS chromatograms of sweet potato peel (SPP; a) and sweet potato flour (SPF; b).
